# ASPP1/2-PP1 complexes are required for chromosome segregation and kinetochore-microtubule attachments

**DOI:** 10.18632/oncotarget.6355

**Published:** 2015-11-22

**Authors:** Pingzhao Zhang, Yuanyuan Zhang, Kun Gao, Yuqi Wang, Xiaofeng Jin, Youheng Wei, Heige Saiyin, Dejie Wang, Jintao Peng, Jian Ma, Yan Tang, Reziya Wumaier, Hongxiu Yu, Yimin Dong, Haojie Huang, Long Yu, Chenji Wang

**Affiliations:** ^1^ State Key Laboratory of Genetic Engineering, Collaborative Innovation Center for Genetics and Development, School of Life Sciences, Fudan University, Shanghai, P.R. China; ^2^ Institutes of Biomedical Sciences, Fudan University, Shanghai, P. R. China; ^3^ Department of Gastroenterology, Jiangxi Institute of Gastroenterology & Hepatology, The First Affiliated Hospital of Nanchang University, Nanchang, Jiangxi Province, P. R. China; ^4^ Department of Urology, Shanghai First People's Hospital, School of Medicine, Shanghai Jiaotong University, Shanghai, P.R. China; ^5^ Department of Pathology, The University of Arizona Medical Center, Arizona, USA; ^6^ Department of Biochemistry and Molecular Biology, Mayo Clinic College of Medicine, Rochester, MN, USA

**Keywords:** chromosome segregation, kinetochore-microtubule attachment, spindle assembly checkpoint, dephosphorylation, protein phosphatase 1, Chromosome Section

## Abstract

Regulated interactions between kinetochores and spindle microtubules are critical for maintaining genomic stability during chromosome segregation. Defects in chromosome segregation are widespread phenomenon in human cancers that are thought to serve as the fuel for tumorigenic progression. Tumor suppressor proteins ASPP1 and ASPP2, two members of the apoptosis stimulating proteins of p53 (ASPP) family, are frequently down-regulated in human cancers. Here we report that ASPP1/2 are required for proper mitotic progression. In ASPP1/2 co-depleted cells, the persistence of unaligned chromosomes and the reduction of tension across sister kinetochores on aligned chromosomes resulted in persistent spindle assembly checkpoint (SAC) activation. Using protein affinity purification methods, we searched for functional partners of ASPP1/2, and found that ASPP1/2 were associated with a subset of kinetochore proteins (Hec1, KNL-1, and CENP-F). It was found that ASPP1/2 act as PP1-targeting subunits to facilitate the interaction between PP1 and Hec1, and catalyze Hec1 (Ser165) dephosphorylation during late mitosis. These observations revealed a previously unrecognized function of ASPP1/2 in chromosome segregation and kinetochore-microtubule attachments that likely contributes to their roles in chromosome stability and tumor suppression.

## INTRODUCTION

Accurate chromosome segregation requires that all sister chromatids are correctly attached to microtubules emanating from opposite poles (bipolar attachment) before sister chromatids separate. Defects in chromosome segregation result in chromosomal instability (CIN) and aneuploidy, which are hallmarks of many cancers [1].

Bipolar attachment of spindle microtubules to kinetochores is monitored by the spindle assembly checkpoint (SAC) and dynamically regulated by phosphorylation to allow correction of improper attachment and stabilization of correct attachments [2]. Multiple mitotic kinases, including Aurora B, Mps1, Bub1, BubR1 and NEK2A, are involved in kinetochore-microtubule attachment by phosphorylating the kinetochore proteins that directly interact with spindle microtubules, including the Ndc80 complex [3], the Dam1 complex [4], and the kinesin-13 family member MCAK [5]. Phosphorylation of substrates at kinetochores destabilizes incorrect attachments, and resets the kinetochore to provide a new opportunity to bi-orient. However, this process requires that substrates are subsequently dephosphorylated to stabilize correct attachments [6]. Protein phosphatase 1 (PP1)-mediated dephosphorylation has emerged as a key regulatory mechanism of this process. In vertebrates, PP1 isoforms α and γ can be detected at outer kinetochores [7, 8], and PP1 has been shown to stabilize kinetochore-microtubule attachment by counteracting Aurora B kinase activity [2]. The puriﬁed catalytic subunit of PP1 has a rather broad substrate speciﬁcity *in vitro*. PP1 specificity *in vivo* is mainly achieved through association of a catalytic subunit with specific targeting subunits that can drive localization and modulate activity and specificity [6]. Recently, the yeast protein Fin1 and kinetochore protein KNL-1 have been identified to target some PP1 to yeast and vertebrate kinetochores, respectively [2, 9]. Another two PP1-targeting subunits, Sds22 and Repo-Man, stabilize chromosome segregation by counteracting Aurora B on anaphase kinetochores [10].

ASPP1 and ASPP2 are two members of the ASPP (Apoptosis Stimulating Proteins of p53) protein family, which includes iASPP. ASPP1 and ASPP2 stimulate, whereas iASPP inhibits, the pro-apoptotic activities of p53 (as well as family members p63 and p73) [11]. ASPP1 and ASPP2 are important tumor suppressors, and their expressions are dramatically reduced in various types of human tumors [12]. Studies in ASPP2 knockout mouse models revealed that ASPP2 heterozygous mice were prone to spontaneous tumors, which clearly demonstrated the role of ASPP2 as a haploinsufficient tumor suppressor [13, 14]. Despite the well-documented interplay between ASPP1/2 and p53, there has been increasing evidence indicating that ASPP1/2 have p53-independent cellular functions: ASPP2 has been shown to bind the PAR complex protein Par-3 at cell junctions and contribute to the maintenance of polarity [15, 16]. ASPP1/2 can bind active RAS to promote oncogene-induced senescence [17, 18]. ASPP1/2 are Hippo pathway activators through enhancing the nuclear accumulation of YAP/TAZ and YAP/TAZ-dependent transcriptional regulation [19, 20]. However, whether these cellular pathways are important for ASPP1/2-mediated tumor suppression remains poorly understood.

We set out to identify additional factors that may be involved in ASPP1/2-mediated cellular function by isolating ASPP1/2 protein complexes from cells. Unexpectedly, we found that ASPP1/2 associated with a subset of kinetochore proteins. Further studies demonstrated that ASPP1/2 were required for proper mitotic progression and faithful chromosome segregation. We also showed that ASPP1/2 could recruit PP1 to dephosphorylate mitotic Hec1. Our studies thus reveal that ASPP1/2 are novel PP1-targeting subunits that play critical roles in chromosome congression and kinetochore-microtubule attachments, and thereby, provided functional insights into understanding of ASPP1/2-mediated tumor suppression.

## RESULTS

### Identification of ASPP1/2 interactomes in HeLa cells

**Figure 1:**
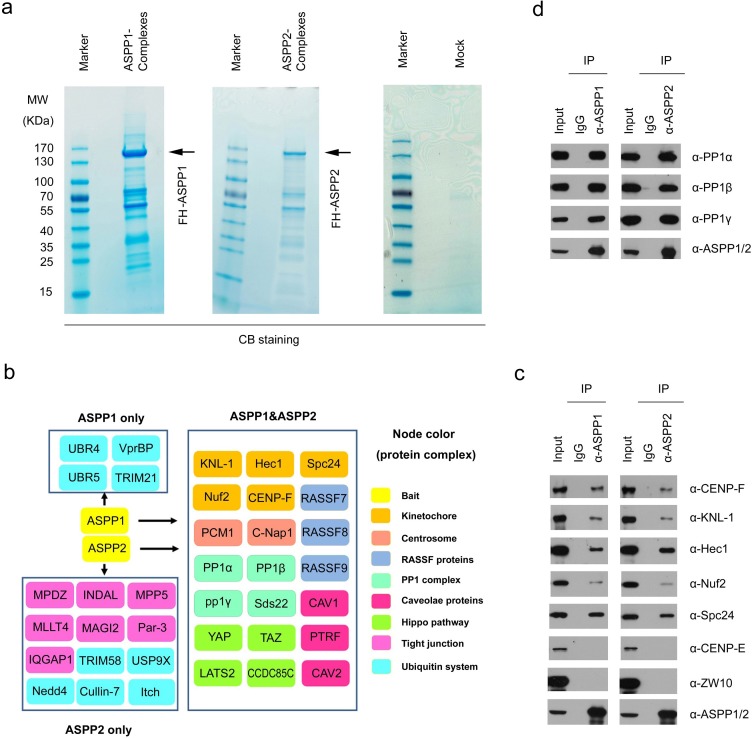
ASPP1/2 interact with multiple kinetochore components **a**. Tandem affinity purification of ASPP1/2-containing protein complexes were conducted using MOCK HeLa cells or cells stably expressing FLAG-HA (FH)-ASPP1 or ASPP2. Associated proteins were separated by SDS-PAGE and visualized by Coomassie Blue(CB)staining. The proteins and the number of peptides identified by mass spectrometry are shown in the Supplementary Table S1, S2. **b**. ASPP1/2-associated protein networks. The ASPP1/2-associated proteins are grouped by functional category (node color/label). **c**. Endogenous ASPP1/2 interact with multiple kinetochore components. Immunoprecipitation with anti-ASPP1 or ASPP2 antibodies were performed using cell lysates prepared from HeLa cells. The presence of kinetochore components in the immunoprecipitates was detected by WB analyses with their indicated antibodies. **d**. Similar to (c), the presence of three PP1 catalytic subunits in the immunoprecipitates was detected by WB analyses with the indicated antibodies.

We isolated ASPP1 and ASPP2 complexes from HeLa cells by Tandem Affinity Purification (TAP) methods and determined the proteins present in these complexes by mass spectrometry. Non-specific binding proteins identified in MOCK HeLa cells were omitted from the list of those identified in FH-ASPP1/HeLa or FH-ASPP2/HeLa cells (Figure 1a and 1b; Table S1 and S2). As veriﬁcation of this approach, many of the known ASPP1/2 binding partners, such as PP1 subunits, Par-3 [15, 16] and Hippo pathway components (YAP1, TAZ, and LATS2) [19, 20], were detected in their complexes. In addition to known interactors of ASPP1/2, other proteins involved in diverse biological processes were co-purified in the ASPP1/2 complexes, including the outer kinetochore proteins (Hec1, KNL-1, Nuf2, Spc24, and CENP-F), centrosome proteins (C-Nap1, and PCM1), RASSF proteins (RASSF7, RASSF8, and RASSF9), and caveolae proteins (CAV1, CAV2, and PTRF) (Figure 1b). In addition, this approach distinguished proteins that may selectively interact with ASPP1 or ASPP2. For example, several ASPP2-speciﬁc binding partners, such as MPDZ, INDAL, MLLT4, MAGI2, and Par-3, are known to be involved in cell tight junction (Figure 1b). Moreover, ASPP1 and ASPP2 seem to have different binding preferences for proteins involved in the ubiquitination process (Figure 1b).

Given that the link between ASPP1/2 and kinetochores has not been reported in the literatures, we aimed to investigate the potential roles of ASPP1/2 in kinetochore biology. We first wanted to confirm whether ASPP1/2 interact with multiple kinetochore proteins. Endogenous immunoprecipitation was performed using cell lysates prepared from HeLa cells. As shown in Figure 1C, Hec1, KNL-1, Nuf2, Spc24, and CENP-F were detected in the anti-ASPP1 or ASPP2 immunoprecipitates by Western blotting (WB). These interactions are specific as we could not detect two other kinetochore proteins (CENF-E and ZW10) in the immunoprecipitates (Figure 1c). Moreover, we confirmed that ASPP1/2 strongly interacted with three PP1 catalytic subunits (α, β, and γ), which were the most abundant ASPP1/2-associated proteins identified by mass spectrometry (Figure 1d).

### Depletion of ASPP1/2 in HeLa cells impaired cell cycle progression

**Figure 2:**
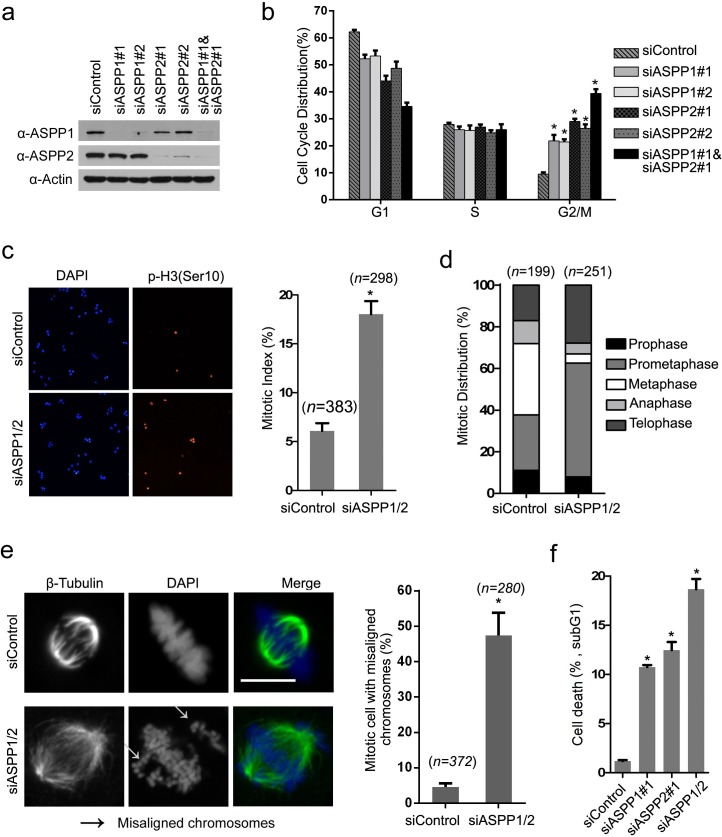
ASPP1/2 are required for proper mitotic progression **a**. Depletion of ASPP1/2 by siRNAs in HeLa cells. HeLa cells were transfected with the indicated siRNAs. After 48 hr, cell lysates were prepared for WB analyses with their indicated antibodies. **b**. ASPP1/2 co-depletion causes G2/M arrest. The cell-cycle distributions of HeLa cells transfected with indicated siRNAs for 48 hr were determined by flow cytometry. Error bars, SEM. *p<0.01 from triplicates. **c**. ASPP1/2 co-depletion increases the mitotic index in HeLa cells. HeLa cells were transfected with control or ASPP1/2 siRNAs as indicated. After 48 hr, cells were fixed and stained for the p-H3 (Ser10) antibody. Quantification of cells with anti-p-H3 (Ser10) staining is shown at the right (n= number of counted cells). **d**. Mitotic stages were quantified by DNA and spindle morphology in the mitotic population of control or ASPP1/2 co-depleted HeLa cells. **e**. ASPP1/2 co-depletion increases the number of mitotic cells with misaligned chromosomes. HeLa cells were transfected with control or ASPP1/2 siRNAs. After 48 hr, cells were fixed and stained with the anti-β-tubulin (green) antibody and DAPI (blue). White arrows indicate misaligned chromosomes. Scale bar = 10μm. Quantification of cells with misaligned chromosomes is shown on the right. **f**. ASPP1/2 depletions lead to increases in cell death. HeLa cells were transfected with control or ASPP1/2 siRNAs. After 72hr, the cell death was measured by flow cytometry using the propidium iodide staining assay.

Considering that ASPP1/2 interacts with several outer kinetochore proteins, we were interested in investigating whether ASPP1/2 have roles in mitosis. In order to determine this, we depleted ASPP1 and ASPP2 individually or in combination in HeLa cells using siRNAs. WB analyses confirmed that ASPP1 and/or ASPP2 protein levels decreased to 10% of control cells at 48 hr after siRNAs transfection (Figure 2a). ASPP1 or ASPP2 depletion using two siRNAs led to an increased number of cells in G2/M phase compared to control cells, as determined by flow cytometry (Figure 2b). Moreover, ASPP1/2 co-depletion caused more increases in number of cells in G2/M phase compared to individual-depleted cells, suggesting ASPP1/2 may have collaborative roles in regulating the cell cycle (Figure 2b). Cytological quantification of mitotic stages, using histone H3 phosphorylated on serine 10 (p-H3Ser10) as a marker of mitosis, confirmed that ASPP1/2 co-depletion significantly increased the mitotic index (Figure 2c), and most of these mitotic cells appeared to be in prometaphase (Figure 2d). Importantly, we observed a 7.25-fold increase of cells with several non-aligned chromosomes around the metaphase plate compared to the control cells (Figure 2e). Aberrant and incomplete mitosis often leads to a type of cell death, called mitotic catastrophe [21]. As expected, we observed that cell death was markedly increased following prolonged ASPP1, or ASPP2 depletion as indicated by the percentage of cells undergoing DNA fragmentation within the sub-G1 phase. ASPP1/2 co-depletion causes more cell death compared to individual-depleted cells (Figure 2f). Moreover, G2/M arrest was also observed in ASPP1/2 co-depleted SMMC-7721(p53 wild-type), HCT116 p53+/+, and HCT116 p53−/− cells (Supplementary Figure S1). Although ASPP1/2 are known as p53 regulators, the results that ASPP1/2 were required for proper mitotic progression both in p53 wild-type and null cells lines suggested that ASPP1/2 may regulate mitotic progression in a p53-indepenent manner. In summary, these results suggested that ASPP1/2 cooperatively regulate mitotic progression, possibly through maintaining proper chromosome segregation.

### Defective kinetochore–microtubule attachments in ASPP1/2 co-depleted cells

**Figure 3:**
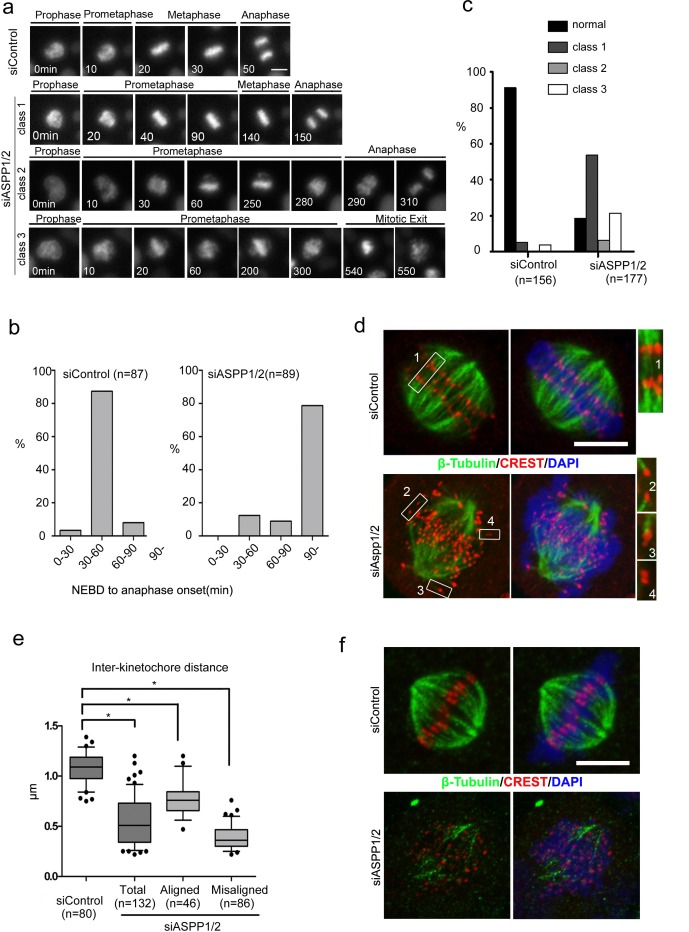
ASPP1/2 are required for proper kinetochore-microtubule attachments **a**. Live-cell imaging of HeLa cells stably expressing H2B-mCherry and transfected with control or ASPP1/2 siRNAs. T= 0 min was defined as the time point at which chromosome condensation became evident (prophase). Scale bar = 10μmm. **b**. Prolonged mitosis in ASPP1/2 co-depleted cells. The time from nuclear envelope breakdown (NEBD) to anaphase onset was measured in live-cell imaging and categorized. The percentages of cells in each category are shown in the graph. **c**. Quantitative analysis of different mitotic phenotypes in control or ASPP1/2 co-depleted cells. **d**. Defective kinetochore–microtubule attachments in ASPP1/2 co-depleted cells. HeLa cells were transfected with control or ASPP1/2 siRNAs for 48 hr, and with MG132 for the final 2 hr. Cells were stained with anti-β-tubulin (red), anti-CREST (green) antibodies, and DAPI (blue). Scale bar = 10 μm. Numbers point to magnified areas and indicate the mode of attachment of k-fibres to kinetochores (1, 2, bi-oriented; 3, mono-oriented kinetochores; 4, unattached). **e**. Quantitative analysis of inter-kinetochore distance in ASPP1/2 co-depleted cells. HeLa cells were treated as in (d). The distance between CREST on sister kinetochores was measured. Error bars, SEM. *p<0.01 from triplicates. f. Instability of kinetochore microtubules in ASPP1/2 co-depleted cells. HeLa cells were treated as in (d), and then incubated on ice for 10 min before fixation. Cells were stained with anti-β-tubulin (red), anti-CREST (green) antibodies, and DAPI (blue). Scale bar = 10 μm.

To examine the mechanism of chromosome missegregation in ASPP1/2 co-depleted cells, we investigated HeLa cells stably expressing Histone H2B-mCherry with live-cell imaging, with or without ASPP1/2 co-depletion. As shown in Figure 3a, in the majority of control cells, mitosis proceeded from nuclear envelope breakdown (NEBD) to anaphase onset in about 30-60 min, with chromosomes perfectly aligned on the metaphase plate (Figure 3a, 3b). In contrast, in ASPP1/2 co-depleted cells, progression from nuclear envelope breakdown (NEBD) to anaphase onset in these cells took much longer (>90 min) than in control cells (Figure 3a, 3b). We observed three main classes of mitotic progression aberration in ASPP1/2 co-depleted cells (Figure 3a, 3c). In the first class, although prometaphase took longer, all the chromosomes ultimately aligned on the metaphase plate and anaphase proceeded normally (Figure 3a). In the second class, anaphase began after a prolonged prometaphase even though some chromosomes had not yet congressed to the metaphase plate (Figure 3a). In the third class with most severe phenotypes, chromosomes failed to align, and after a prolonged prometaphase, the cells exited mitosis without undergoing a clear anaphase (Figure 3a). The relative frequency of these three different classes may depend on the efficiency of ASPP1/2 co-depletion. Moreover, we observed that a large proportion of cells with abnormal mitotic phenotypes underwent cell death, characterized by membrane blebbing and cell shrinkage (data not shown), which was consistent with results of sub G1 analysis by flow cytometry.

To address the underlying cause of chromosome missegregations observed in ASPP1/2 co-depleted cells, cells were treated with MG132 and examined by immunoﬂuorescence staining. In control cells, each pair of kinetochores on sister chromatids were aligned on the metaphase plate and were pulled towards opposite spindle poles with robust K-ﬁbres (Figure 3d, inset 1). In ASPP1/2 co-depleted cells, while some chromosomes were correctly aligned (Figure 3d, inset 2), many chromosomes were not aligned on the metaphase plate (Figure 3d, inset 3, 4). However, kinetochores on misaligned chromosomes were still paired, indicating that precocious separation of sister chromatids had not occurred. These misaligned sister chromatid pairs often attached to only one kinetochore, or did not attach at all to the microtubules (Figure 3d, insets 3 and 4). Defects in chromosome alignment suggested impairment of chromosome attachments or failure to correct attachment errors. The tension exerted by microtubules on properly attached kinetochores increases the distance between sister kinetochores. The inter-kinetochore distance in ASPP1/2 co-depleted cells was shorter than in control cells, for both misaligned and aligned chromosomes (Figure 3e), suggesting that tension was not properly exerted between the sister kinetochores. To examine the stability of kinetochore–microtubule attachments, MG132-treated cells were exposed to low temperatures, which induced the disassembly of unstable microtubules. In control cells, thick K-ﬁbres were clearly attached to each kinetochore, whereas in ASPP1/2 co-depleted cells, few cold-stable K-ﬁbres were observed (Figure 3f). In summary, these results suggested that ASPP1/2 co-depletion resulted in defective kinetochore-microtubule attachments, which caused mitotic delay and subsequent collapse of the metaphase plate.

### Co-depletion of ASPP1/2 in HeLa cells causes SAC hyperactivation

**Figure 4:**
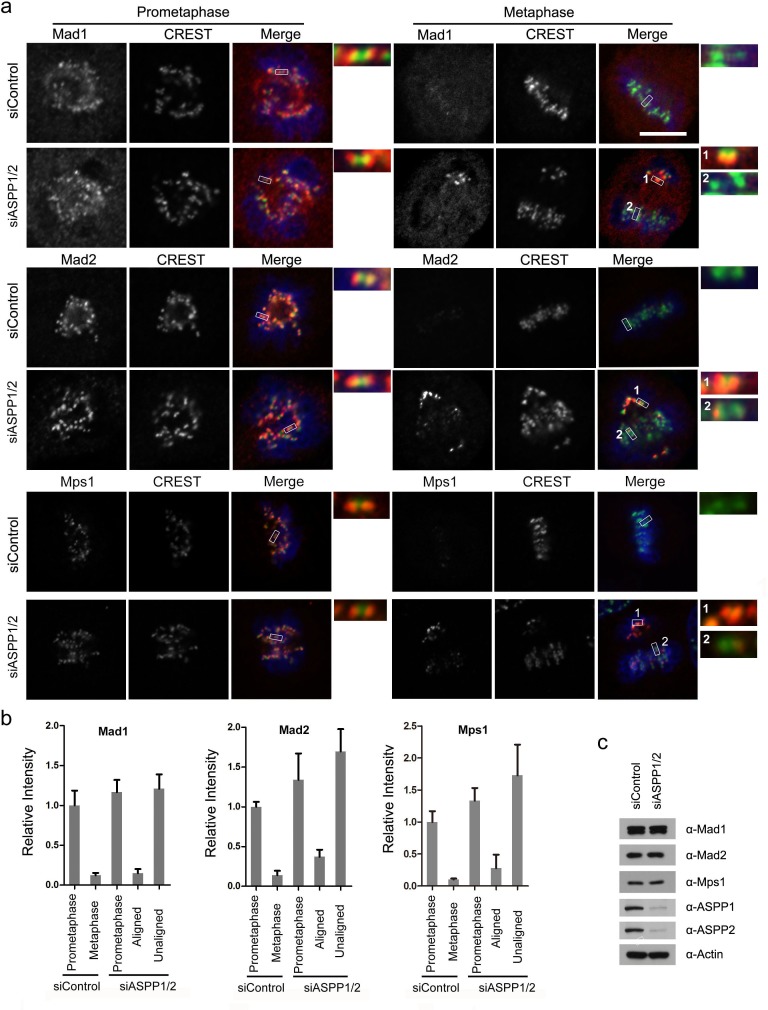
ASPP1/2 co-depletion causes SAC hyperactivation **a**. Localization of Mad1, Mad2 and Mps1 in ASPP1/2 co-depleted HeLa cells. HeLa cells were transfected with control or ASPP1/2 siRNAs for 48 hr, and treated with nocodazole for 12 hr and then released into fresh media for 1-2 hr before fixation. Cells were stained with antibodies against the indicated SAC proteins (red), together with kinetochores (CREST, green) and DNA (blue). The figures show confocal images of cells at prometaphase and metaphase. Insets are magnified images of the boxed areas. Scale bar = 10 μm. **b**. Quantification of the fluorescence intensity of the SAC proteins normalized to the fluorescence intensity of CREST staining are shown. For quantifications, ∼30 mitotic cells were measured for each experiment and condition. Error bars, SEM *p<0.01 from triplicates. c. WB analyses of cell lysates prepared from control and ASPP1/2 co-depleted HeLa cells using the indicated antibodies.

Because ASPP1/2 co-depleted cells at metaphase contains kinetochores that are unattached or under partial tension, we investigated the state of the SAC. In control cells, the SAC proteins Mad1, Mad2 and Mps1, which monitor attachment, were localized to kinetochores at prometaphase but disappeared at metaphase (Figure 4a, 4b). As expected, kinetochores on unaligned chromosomes in ASPP1/2 co-depleted cells exhibited much higher Mad1, Mad2 and Mps1 levels than that on aligned chromosomes (Figure 4a, 4b). Importantly, ASPP1/2 co-depletion did not affect the overall expression levels of Mad1, Mad2 and Mps1 (Figure 4c). In summary, these results suggest that in the absence of ASPP1/2, SAC signaling on kinetochores was activated due to a lack of proper attachment.

### ASPP1/2 facilitates the interaction between Hec1 and PP1α

**Figure 5:**
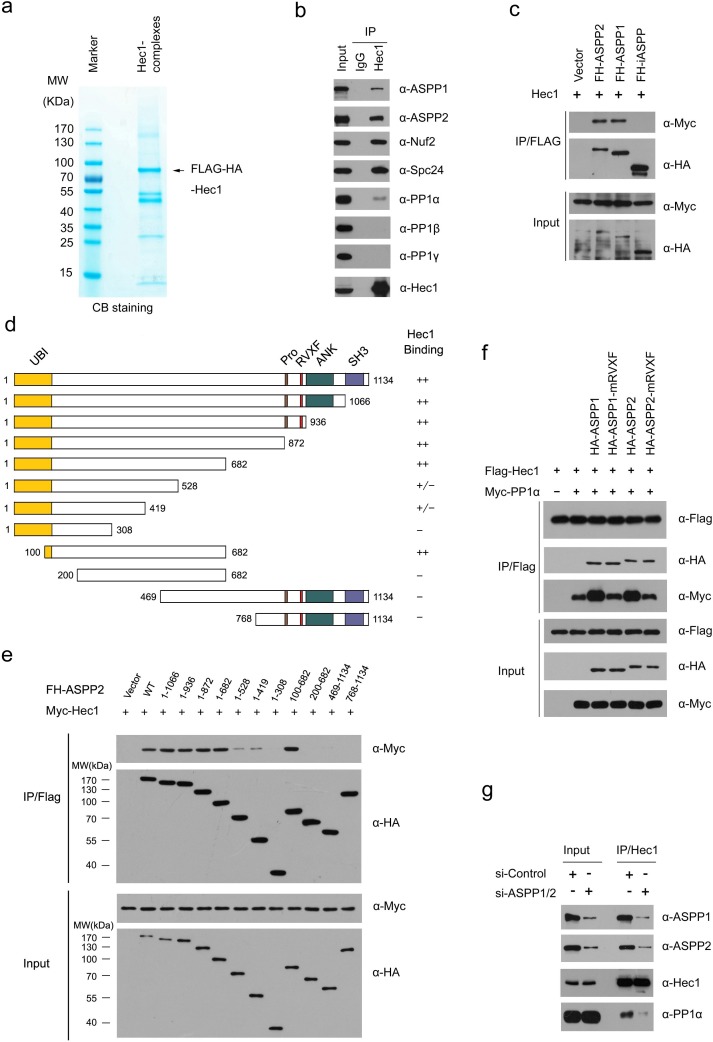
ASPP1/2 facilitate the interaction between Hec1 and PP1α **a**. Tandem affinity purification of the Hec1-containing protein complex was conducted using HeLa cells stably expressing FLAG-HA (FH)-Hec1. Associated proteins were separated by SDS-PAGE and visualized by CB staining. The proteins and the number of peptides identified by mass spectrometry analysis are shown in the Supplementary Table S3. **b**. Endogenous Hec1 interaction with ASPP1/2 and PP1α. Immunoprecipitation with anti-Hec1 antibody was performed using cell lysates prepared from HeLa cells. The presence of proteins in the immunoprecipitates was detected by WB analyses using the indicated antibodies. **c**. iASPP cannot interact with Hec1. 293T cells were co-transfected with Myc-Hec1 and FH-ASPP (ASPP1, ASPP2 or iASPP) constructs. After 24 hr, cell lysates were prepared for immunoprecipitation with the anti-Flag antibody and detected by WB analyses using the indicated antibodies. **d**. Schematic representation of ASPP2 deletion mutants. Binding capacity of ASPP2 WT or mutants to Hec1 is indicated with the symbols. **e**. Identification of Hec1-binding domain in ASPP2. 293T cells were co-transfected with Myc-Hec1 and FH-ASPP2-WT or deletion mutants. After 24 hr, cell lysates were prepared for immunoprecipitation with anti-FLAG antibody and detected by WB analyses. **f**. ASPP1/2 facilitate the interaction between Hec1 and PP1α in a PP1-binding dependent manner. 293T cells were co-transfected with indicated constructs. After 24 hr, cell lysates were prepared for immunoprecipitation with the anti-Flag antibody and detected by WB analyses using indicated antibodies. **g**. ASPP1/2 co-depletion reduces the endogenous interaction between Hec1 and PP1α. HeLa cells were transfected with the control or ASPP1/2 siRNAs. After 48 hr, cell lysates were prepared for immunoprecipitation with anti-Hec1 antibody and detected by WB analyses using the indicated antibodies.

Next, we explored the underlying molecular mechanisms of ASPP1/2 in controlling kinetochore-microtubule attachment. Among ASPP1/2-associaicted kinetochore proteins, Hec1 is the core component of the Ndc80 complex that plays critical roles in assembling kinetochores and functions to congress chromosomes and to signal the spindle assembly checkpoint [22, 23]. To further elucidate the functional relationship between ASPP1/2 and Hec1, we first isolated the Hec1 complex from HeLa cells and determined the proteins present in the complex by mass spectrometry (Figure 5a; Supplementary Table S3). In addition to known Ndc80 complex components such as Nuf2 and Spc24, ASPP1/2 were also identified as major Hec1-associated proteins (Figure 5b). Moreover, co-immunoprecipitation assay showed that iASPP, another member of the ASPP family, did not interact with Hec1, suggesting that ASPP1/2-Hec1 interactions are highly specific (Figure 5c). Interestingly, although we demonstrated ASPP1/2 interact with kinetochore proteins using biochemical methods, immunofluorescence staining showed no obvious kinetochores localization of endogenous or mCherry-ASPP1/2 in mitotic stages using diverse fixation methods (Supplementary Figure S2). One possible explanation of this observation is that the ASPP1/2-Hec1 interaction in kinetochores may be transient and dynamic. The detailed mechanism of this phenomenon is still being explored.

ASPP1/2 proteins show 60% sequence similarity, and possess a similar modular structure, including an Ubiquitin-like domain (Ubl), ankyrin domain (ANK), SH3 domain, and Pro-rich domain (Pro) (Figure 5d). To determine which domain mediates its interaction with Hec1, we performed a co-immunoprecipitation assay with Hec1 and a series of deletion mutants of ASPP2. As shown in Figure 5d and 5e, the region corresponding to 100-682 aa of ASPP2, which does not contain any known structural motifs, is responsible for Hec1 binding. This interaction pattern is distinct from that of other ASPP2 interactors, such as p53, Bcl-2, and p65-NFκB, which bind to the C-terminal part (ANK-SH3 domains) of ASPP2 [24].

We sought to determine whether the kinetochore localization of Hec1 was affected in ASPP1/2 co-depleted cells. However, no obvious change in the kinetochore localization or protein level of Hec1 was detected in ASPP1/2 co-depleted cells compared to control cells (Supplementary Figure S3). Thus, we hypothesized that ASPP1/2 may affect Hec1 interactions with other proteins. Our mass spectrometry results showed that PP1α, but not PP1β or PP1γ, was co-purified with Hec1 complexes (Supplementary Table S3), and the specific interaction between endogenous Hec1 and PP1α, but not PP1β or PP1γ was verified by WB analyses (Figure 5b). Since previous study showed ASPP2 can facilitate the interaction between TAZ and PP1α to promote TAZ dephosphorylation at Ser89 and Ser311 [19], we investigated whether ASPP1/2 act as molecular adaptors to facilitate the interaction between Hec1 and PP1α. As expected, a co-immunoprecipitation assay showed that co-expression of ASPP1/2 markedly increased the interaction between Hec1 and PP1α (Figure 5f). ASPP1/2 have a conserved PP1-binding motif (RVXF) near the central region [25]. To test whether the interaction with PP1α is important for the roles of ASPP1/2 in the enhancement of Hec1-PP1α interaction, we made ASPP1/2 mRVXF mutants that carried three substitutions in each of the conserved motifs (RVXF-AAxA). As expected, the ASPP1/2 mRVXF mutants lost their ability to enhance Hec1-PP1α interaction (Figure 5f). In agreement with the above findings, ASPP1/2 co-depletion significantly reduced the interaction between endogenous Hec1 and PP1α (Figure 5g). In summary, these results suggested that ASPP1/2 can facilitate the interaction between Hec1 and PP1α in a PP1-binding dependent manner.

### ASPP1/2-PP1 complexes dephosphorylate mitotic Hec1 at Ser165

Next, we investigated whether ASPP1/2 can modulate the mitotic phosphorylation of Hec1 in cellular models. Hec1 undergoes extensive phosphorylation at multiple sites by mitotic kinases, including Aurora B, Mps1 and NEK2A [26-29]. Studies in yeast and human cells showed that mimicking Ndc80 phosphorylation triggers SAC hyperactivation, suggesting that Ndc80 dephosphorylation is required for SAC silencing and mitotic exit [29, 30]. To investigate whether ASPP1/2 were required for mitotic exit, cell lysates were prepared from HeLa cells synchronized in prometaphase and then released to fresh media to allow the completion of mitosis. As shown in Supplementary Figure S4, Securin and Cyclin B1 degradation, and phospho-H3 (Ser10) dephosphorylation, were greatly compromised in ASPP1/2 co-depleted cells, suggesting that ASPP1/2 are indispensable for mitotic exit.

**Figure 6:**
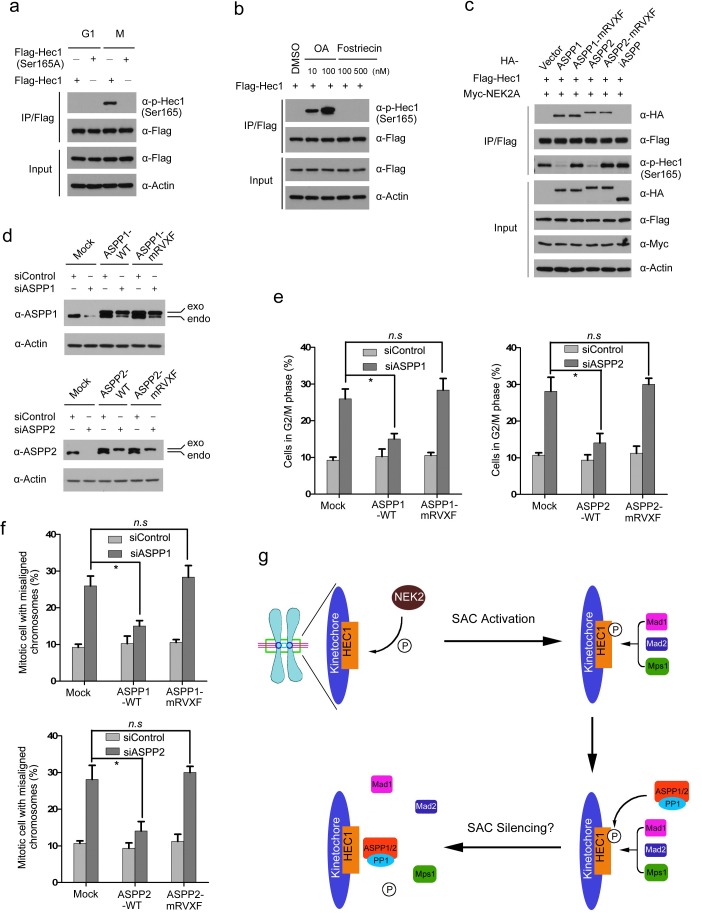
ASPP1/2-PP1 complexes dephosphorylate mitotic Hec1 at Ser165 **a**. HeLa cells were transfected with Flag-Hec1 WT or S165A mutant. Cells were synchronized at the G1/S (double-thymidine block) or M phase (nocodazole). Cell lysates were prepared for immunoprecipitation with the anti-Flag antibody and detected by WB analyses using the indicated antibodies. **b**. 293T cells were transfected with Flag-Hec1. After 24hr, the cells were treated with different doses of Okadaic acid (OA) or Fostriecin for another 12hr. Cell lysates were prepared for immunoprecipitation with the anti-Flag antibody and detected by WB analyses using the indicated antibodies. **c**. ASPP1/2 antagonize NEK2A-mediated Hec1 Ser165 phosphorylation. 293T cells were co-transfected with indicated constructs. After 24 hr, cell lysates were prepared for immunoprecipitation with the anti-Flag antibody and detected by WB analyses using the indicated antibodies. **d**. WB analyses of ASPP1/2 proteins following siRNA treatment in HeLa cells stably expressing a FH-ASPP1/2 (WT or mRVXF) constructs resistant to the siRNAs targeting endogenous ASPP1/2. (**e**., **f**.) Stable expression of siRNA-insensitive FH-ASPP1/2, but not the mRVXF mutants, in siRNA-treated HeLa cells rescued G2/M arrest (e) chromosome misalignment (f) caused by ASPP1/2 depletion. The cell-cycle distributions of HeLa cells transfected with the indicated siRNAs for 48 hr were determined by flow cytometry. Error bars, SEM *p<0.01 from triplicates. *n.s*, not statistically significant. (f) Model.

We explored whether ASPP1/2 were involved in Hec1 dephosphorylation. Previous studies demonstrated that NEK2A-mediated Ser165 phosphorylation of Hec1 was critical for proper chromosome-microtubule attachment and the SAC signal. ^30^ The phospho-Hec1 (Ser165) signal diminished at metaphase kinetochores when chromosomes had achieved proper alignment, correlating with SAC silencing. Importantly, inhibition of PP1 preserved the phospho-Hec1 (Ser165) signal, suggesting that dephosphorylation at this site is regulated by PP1 [30]. However, considering that the PP1 holoenzyme does not exhibit strong substrate selectivity, how PP1 is specifically recruited to dephosphorylate phospho-Hec1 (Ser165) remains obscure. To test whether ASPP1/2 regulate the dephosphorylation of phospho-Hec1 (Ser165), we generated a phospho-Hec1 (Ser165) antibody which specifically recognized ectopically expressed wild-type Hec1, but not Hec1 Ser165A at mitosis (Figure 6a). Moreover, the phospho-Hec1 (Ser165) signal was greatly enhanced by treating cells with Okadalic acid (PP1 inhibitor), but not with the Fostriecin (PP2A inhibitor) (Figure 6b). Next, ASPP1/2 (WT or mRVXF), Hec1 and NEK2A constructs were co-expressed in 293T cells. Hec1 was immunoprecipitated and the phospho-Hec1 (Ser165) signal was detected by WB using a phosphorylation-specific antibody. As shown in Figure 6c, co-expression with ASPP1 or ASPP2-WT dramatically reduced the NEK2A-mediated Hec1 Ser165 phosphorylation, whereas these effects were not observed by co-expression with ASPP1/2-mRVXF mutants or iASPP. Our results also showed dephosphorylation of phospho-Hec1 (Ser165) was greatly compromised during mitotic exit in ASPP1/2 co-depleted cells (Supplementary Figure S4). Thus, these results suggest that ASPP1/2 can antagonize NEK2A-mediated Hec1 Ser165 phosphorylation in a PP1 binding-dependent manner.

To further investigate if ASPP1/2-mediated cell cycle regulation is dependent on their PP1-binding ability, HeLa cells stably expressing siRNA-insensitive -ASPP1/2 (WT or mRVXF) were generated (Figure 6d). When the endogenous ASPP1 was depleted by siRNAs, signiﬁcant rescue of G2/M arrest and chromosome misalignment were observed in HeLa cells stably expressing ASPP1-WT, whereas these effects were not observed in HeLa cells stably expressing ASPP1-mRVXF (Figure 6e and 6f). Similar phenotypes were observed in ASPP2 stable cell lines when endogenous ASPP2 was depleted (Figure 6e and 6f). In conclusion, our results suggest that ASPP1/2 can promote Hec1 dephosphorylation, at least at Ser165. Moreover, the PP1 binding capability of ASPP1/2 seems indispensable for their function in cell cycle regulation.

## DISCUSSION

The data presented here revealed ASPP1/2 as two PP1-targeting subunits that play critical roles in mitotic exit. We also demonstrated that Hec1 was one of the kinetochore substrates regulated by ASPP1/2-PP1 complexes during mitosis. Therefore, our findings suggest that a possible pathway may exist through which ASPP1/2 under-expression promote SAC hyperactivation, chromosome missegregation and aneuploidy. Coordinated mitotic regulation by ASPP1/2-PP1 complexes could be critical for a number of physiological functions that prevent cancer.

SAC proteins are recruited to kinetochores in a hierarchical manner with the Mps1 checkpoint kinase required for the localization of all downstream components, including Bub1-Bub3 and Mad1-mad2 complexes [31]. Two recent studies showed that Mps1 directly binds to the Ndc80 complex and interacts strongly with the Hec1 CH domain [32, 33]. Importantly, binding of microtubules to Hec1 *in vitro* and in cells prevents Mps1 binding showing direct competition. Furthermore, phosphorylation of the N-terminal tail of Hec1 increases the affinity for Mps1 while preventing microtubule binding [32, 33]. Previous studies showed Hec1 Ser165 phosphorylation triggers Mad1/Mad2 recruitment to the kinetochore [30]. phospho-Hec1 (Ser165) has to be removed to allow timely anaphase entry; failure to remove phospho-Hec1 (Ser165) lead to massive segregation errors and robust SAC signaling [30]. Therefore, we proposed a model that, when correct kinetochore-microtubule attachment was established at metaphase, ASPP1/2-PP1 complexes were recruited to remove phospho-Hec1 (Ser165) signal. This leads to dissociation of SAC proteins from kinetochores and triggers timely anaphase entry (Figure 6g). However, further works were needed to clarify whether ASPP2-PP1 complexes play direct roles in SAC silencing or it is indirect through regulating kinetochore-microtubule attachment.

In addition to ASPP/2, several PP1-targeting subunits, including KNL-1 and Sds22 are implicated in Aurora B regulation at the kinetochores [6]. For example, Sds22 can recruit PP1 to kinetochores to counteract Aurora B–dependent phosphorylation of the outer kinetochore protein Dsn1 during mitosis [10]. Sds22 depletion induces a high incidence of chromosome missegregation similar to ASPP1/2 co-depletion [10]. KNL-1 is a member of the conserved KMN (KNL-1/Mis12 complex/Ndc80 complex) network of kinetochore proteins. A RVXF motif in the KNL-1 directly interacts with and targets PP1 to the outer kinetochores. PP1 recruitment by KNL-1 is required to dephosphorylate Aurora B substrates at kinetochores and stabilize microtubule attachments [2]. Therefore, it is possible that multiple PP1-targeting subunits act concurrently to remove the mitotic kinases-mediated phosphorylation of kinetochore components when chromosomes are aligned at metaphase in order to allow timely anaphase onset and mitotic exit. Currently, we did not know why no obvious kinetochores localization of ASPP1/2 was observed in mitotic stages, but we noticed it may be not the sole case, as a recent study also showed Sds22 does not normally localize to kinetochores [34]. Instead, Sds22 is kept in solution by formation of a ternary complex with PP1 and inhibitor-3(IPP3). Depletion of IPP3 does not affect the amount of PP1 at kinetochores but causes quantitative association of Sds22 with PP1 on KNL-1 at the kinetochores [31]. Therefore, how ASPP1/2 are dynamically localized to kinetochores still need further study.

In addition to NEK2A, previous reports showed that Hec1's affinity with microtubule is also under the regulation of Aurora B and Mps1 kinases during mitosis [27, 29]. Phosphorylation at multiple sites of the Hec1 N-terminus by Aurora B strongly destabilizes the kinetochore-microtubule attachments [27]. Each of these sites is highly phosphorylated in early mitosis and phosphorylation significantly decreases as chromosomes bi-orient [35]. Moreover, at least ten Ser/Thr sites in the N-terminal domain of Hec1 (1-257) can be phosphorylated yeast Mps1 [29]. Whether ASPP1/2-PP1 complexes are involved in the removal of Aurora B or Mps1-mediated Hec1 phosphorylation at late mitosis was not investigated in this study due to the lack of phosphorylation site-specific antibodies, but it needs to be addressed in further studies. We also noticed that two other phosphatases, PPM1B and PP2A regulatory subunit B55α are co-purified with the Hec1 protein complex (Supplementary Table S3). Interestingly, a recent live-cell imaging RNAi screen identified the PP2A-B55α complex as a key mitotic exit regulator in human cells [36], raising the possibility that Hec1 may also be dephosphorylated by the PP2A-B55α complex at some sites during late mitosis. We therefore hypothesized that the dynamic regulation of Hec1 phosphorylation at mitosis may be controlled by the precise balance between multiple kinases and phosphatases.

Although our findings implicate the role of ASPP1/2 via interactions with Hec1 and promote its dephosphorylation, it is also possible that ASPP1/2 participate in mitotic regulation through regulating other kinetochore partners, such as KNL-1 and CENP-F. Previous study demonstrated that KNL-1 Ser24 and Ser60 are phosphorylated at kinetochores by Aurora B to fine tune kinetochore-microtubule attachments [37]. Moreover, phosphorylation of the conserved MELT motifs in KNL-1 by Mps1 kinase recruits Bub1 and Bub3 to the kinetochores and this is required to maintain the SAC signal [38, 39]. It would be interesting to investigate whether ASPP1/2-PP1 complexes antagonize Aurora B or Mps1-mediated KNL-1 phosphorylation. CENP-F is a component of the nuclear matrix required for chromosome congression that, at mitotic entry, localizes to the nuclear envelope and assembles on kinetochores, contributing to the establishment of kinetochore-MT attachments [40]. Although no literature has demonstrated that CENP-F was phosphorylated and regulated by specific kinases, several quantitative phosphoproteomics studies using HeLa cells in different mitotic stages showed that CENP-F undergoes extensive phosphorylation at numerous sites, and some of these phosphorylation sites are dynamically regulated during mitosis (http://www.phosphosite.org). Like Hec1, our results showed that no obvious change in the kinetochore localization or protein level of KNL-1 or CENP-F was detected in ASPP1/2 co-depleted cells compared to control cells (Supplementary Figure S3). We speculate that KNL-1 and CENP-F are two potential candidates dephosphorylated by ASPP1/2-PP1 complexes during late mitosis, but emphasize that the detailed mechanism remains to be determined. Moreover, our recent results showed that ASPP1/2 interacts with C-Nap1 and antagonize NEK2A-mediated C-Nap1 phosphorylation, which was required for centrosome linker reassembly during late mitosis [41]. PP1α and PP1γ were previously found to directly interact with NEK2A and shown to counteract NEK2A activation [42-44]. Thus, it is also possible that ASPP1/2 inactivate NEK2A activity by enhancing PP1α and PP1γ-mediated dephosphorylation of NEK2A. Collectively, our studies suggest that the ASPP1/2-PP1 complexes may be implicated in multiple organelle dynamics during mitosis by modulating the phosphorylation status of various substrates.

## MATERIALS AND METHODS

### Cell culture

293T and HeLa cells were obtained from the American Type Culture Collection (ATCC). SMMC-7721 cells were obtained from the Cell Bank of the Chinese Academy of Sciences (CAS). HCT116 p53+/+ and HCT116 p53−/− cells were generous gifts from Dr. Bert Vogelstein (The Johns Hopkins University). The cells were maintained in DMEM with 10% (v/v) FBS. All cells were grown at 37°C with 5% CO_2_.

### Plasmids constructions

The ASPP2 cDNAs was kindly provided by Dr. Xin Lu (University of Oxford). The ASPP1, iASPP, and Hec1 cDNAs were obtained from Genecopoeia Inc. All the cDNAs were subcloned into pCIN4-FLAG–HA and pCMV-HA/Myc vectors. The PP1α cDNAs was kindly provided by Dr. Qunyin Lei (Fudan University) and subcloned into pCMV-Myc vectors. ASPP1-mRVXF, ASPP2-mRVXF constructs were generated by the KOD-Plus Mutagenesis Kit (TOYOBO). To generate the RNAi-insensitive cDNAs for ASPP1 or ASPP2, the wobble codons corresponding to the siRNA oligos of ASPP1 or ASPP2 were mutated.

### RNA interference and rescue

For siRNA treatments, cells were transfected using Lipofectamine RNAiMAX (Invitrogen) and 0.05 μM siRNA oligos. The siRNA oligos were purchased from Genepharma Inc: siASPP1#1 (GCUCAUGGAAGAUCCAAAU), siASPP1#2 (CCCGAACUAUGUUGGAAAU), siASPP2#1 (AAGUUGCUGAGCAGGAGAAA), siASPP2#2 (UAUGCAGAGACGUGGUGGA), and siControl (ACAGACUUCGGAGUACCUG). To rescue the defects caused by ASPP1 (or ASPP2) depletion, Stable cells lines that express RNAi-insensitive FH-ASPP1 (or ASPP2) similar with endogenous ASPP1 (or ASPP2) levels were used for siRNA treatments.

### Antibodies

Antibody specific to phospho-Ser165 of Hec1 was raised against the synthetic phosphopeptide CKRIFKDLGTPFAL(pS)KSSM. Phospho-specific antibody was obtained though two-step affinity-purification methods. Commercially available antibodies for WB were as follows: ASPP1 (ab137537; Abcam), ASPP2 (611354; BD Biosciences), Hec1 (ab3613; Abcam), Hec1 (3622-1; epitomics), α-Tubulin (1878-s; epitomics), β-Tubulin (05-661; Millipore), KNL-1 (NB100-2586; Novus), ZW10 (ab21582; Abcam), CENP-E (ab5093; Abcam), CENP-F (ab5093; Abcam), Mad1 (sc-67338; Santa Cruz), Mad2 (7938-1; epitomics), MPS1 (05-682; EMD Millipore), PP1α (1950-1; epitomics), PP1β (2029-1; epitomics), PP1γ (6646-1; epitomics), Cyclin B1 (1495-1; epitomics), CREST (15-235-0001; antibodies incorporated), p-H3(Ser10) (Sc-8656-R; Santa Cruz), Secruin (2603-1; epitomics), Myc (9E10; Sigma), Flag (M2; Sigma), HA (MM5-101R; Millipore) and Actin (AC-74; Sigma).

### Flow cytometry analysis

Flow cytometry analysis was performed and analyzed by flow cytometry (FACSCalibur, BD Biosciences) following cell DNA staining with propidium iodide (PI). Briefly, 1×10^6^ cells were harvested and suspended with ice-cold 70% ethanol, then fixed at −20°C for at least 2 hr. Following harvesting and washing, cells were stained with 0.5 ml of propidium iodide (10μg/ml) and RNase (100μg/ml) in PBS for 30 min at room temperature in the dark and then submitted to flow cytometry analysis. For cell apoptosis analysis, cells were harvested and washed, followed by propidium iodide staining (10μg/ml) with 0.03% triton permeation and RNase treatment. Results are representatives of three independent experiments with triplicate samples for each condition.

### Protein complex purification

The epitope-tagging strategy to isolate ASPP1 (or ASPP2)-containing protein complexes from human cells was performed essentially as previously described with some modifications [45]. In brief, to obtain a FLAG-HA-ASPP1 (or ASPP2) expressing cell line, HeLa cells were transfected with pCIN4-FLAG-HA-ASPP1 (or ASPP2) constructs and selected for 2 weeks in 1 mg/ml G418. The tagged ASPP1 or ASPP2 protein levels were detected by WB analyses. The stable cell lines were chosen to expand for protein complex purification. For purification, the MOCK, HeLa/ASPP1, or HeLa/ASPP2 cells were lysed in BC100 buffer (20 mM Tris-Cl, pH 7.9, 100 mM NaCl, 0.2 mM EDTA, 20% glycerol) containing 0.2% Triton X-100 and fresh protease inhibitor on ice for 2 hr. The homogenate was centrifuged for 30 min at 12000 rpm at 4°C. Cleared lysates were filtered through 0.45 μM spin filters (Millipore) and immunoprecipitated by anti-FLAG antibody-conjugated M2 agarose (Sigma). The bound polypeptides eluted with the FLAG peptide (Sigma) were further affinity purified by anti-HA antibody-conjugated agarose (Sigma). The final elutes from the HA-beads with HA peptides were resolved by SDS-PAGE on a 4%–20% gradient gel (Bio-Rad) for Coomassie Blue staining. Gel bands were cut out from the gel and subjected to mass-spectrometric sequencing.

### Immunofluorescence, confocal microscopy and live cell imaging

For immunofluorescence, cells were plated on chamber slides, fixed either with methanol at −20°C for 5 min or with 4% paraformaldehyde at 37°C for 15min depending on the antibodies used. To examine the protein levels at each mitotic stage, cells were synchronized by double-thymidine block and release to fresh media for various times. A staging system was used to identify the different phases of mitosis and cytokinesis based on the DNA and spindle morphology and extent of chromosome alignment and separation. To test the stability of Microtubule capture at kinetochores, cells were incubated for 5 min on ice before fixation, to destabilize most non-kinetochore Microtubules. After fixation, cells were permeabilized with 0.2% Triton for 5 min, preincubated with centrifuged (14000 rpm) supernatant of 5% FBS and 5% goat serum in PBS and incubated with primary antibodies overnight. Slides were washed, incubated with fluorescence-tagged secondary antibodies and counterstained with DAPI for 1 hr at 4°C. Cells were visualized and imaged using a confocal microscope. Images of proteins of interest as well as CREST on kinetochores were acquired by using identical imaging settings. For quantifying kinetochore intensities using Image J, a circular region with fixed diameter was centered over the kinetochore and intensities were measured for both the protein of interest and for CREST. CREST was used for normalization after subtraction of background intensity. Experiments were carried out with three or more replicates unless otherwise stated. Statistical analyses were performed by Student's t-test for most studies. Values with *p <0.01 are considered statistically significant.

Live cell imaging was performed using HeLa cells stably expressing mCherry-H2B. Cells were transfected with control or ASPP1/2 siRNAs, and then planted in 2-cm dishes with a cover glass window in the glass bottom. Imaging was performed using a confocal (Zeiss LSM 710) and Observer.Z1 (Zeiss) microscope for 24hr. A minimum of 10 movies were scored for each experimental condition.

## SUPPLEMENTARY MATERIAL FIGURES AND TABLES




