# Supplementary material for: ASPP1/2-PP1 complexes are required for chromosome segregation and kinetochore-microtubule attachments
Source: Oncotarget. 2015 Nov 22;6(39):41550–65. doi: 10.18632/oncotarget.6355 (PMC4747173; doi:10.18632/oncotarget.6355)
Supplement: Supplementary file 1 [file oncotarget-06-41550-s001.pdf]

ASPP1/2-PP1 complexes are required for chromosome segregation and kinetochore-microtubule attachments

Supplementary Material

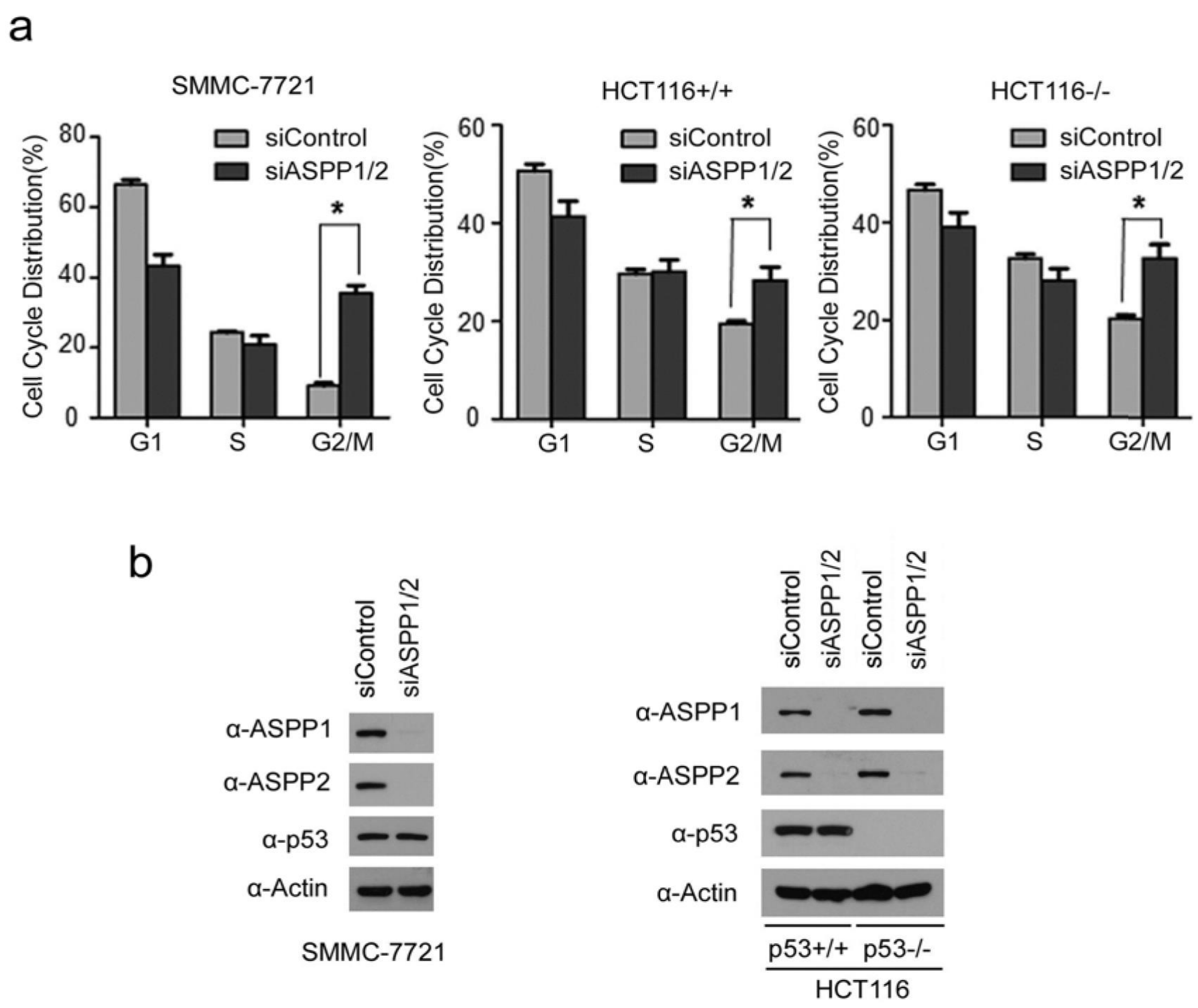

Figure S1. ASPP1/2 co-depletion causes G2/M arrest in p53 wild-type and null cells. (**a**) The cell-cycle distributions of SMMC-7721, HCT116 p53+/+ and HCT116 p53-/-cells transfected with the indicated siRNAs for 48 hr were determined by flow cytometry. Error bars, SEM. *p<0.01 from triplicates. (**b**) WB analyses of the cell lysates prepared from control and ASPP1/2 co-depleted HeLa cells with indicated antibodies.

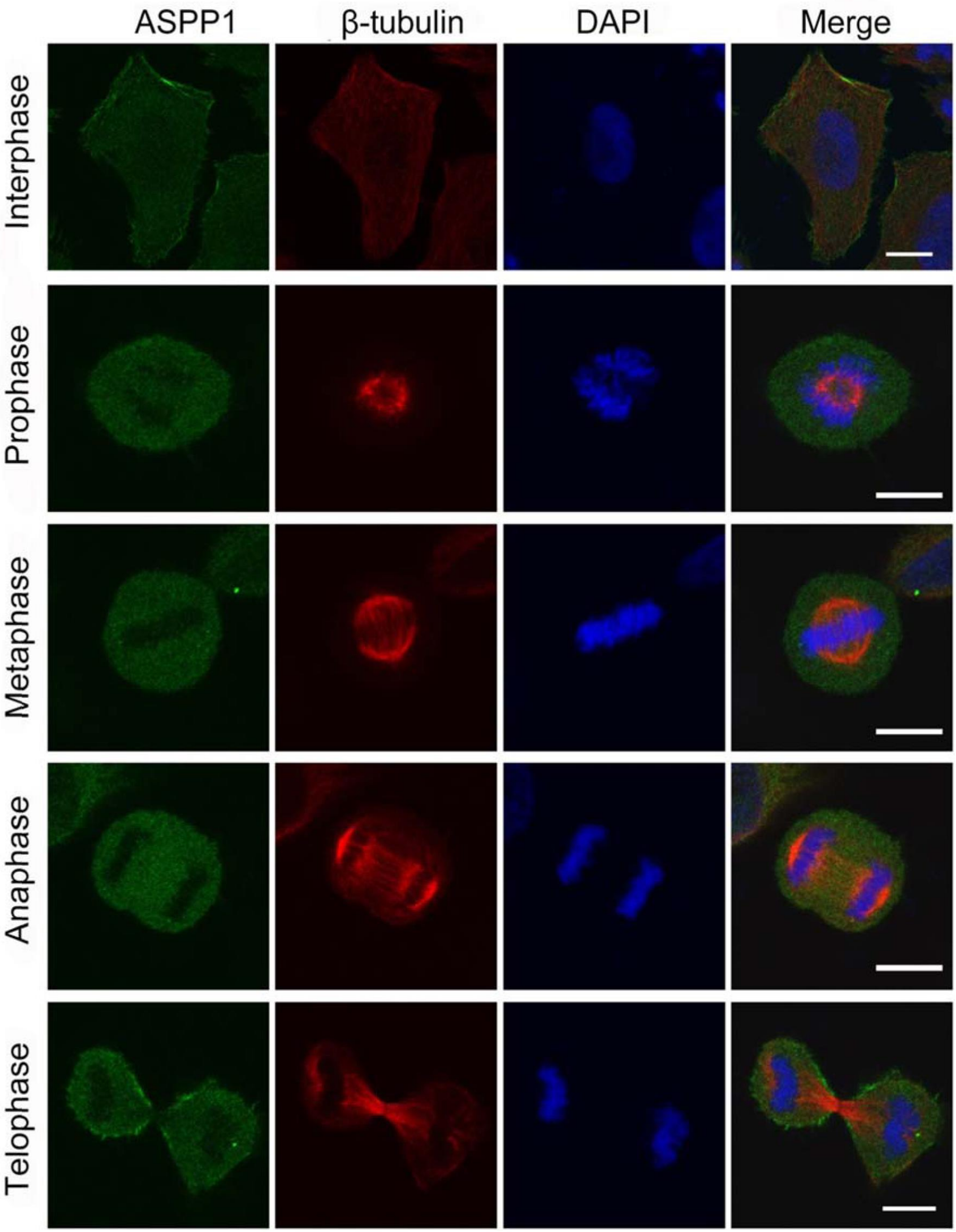
ASPP1
β-tubulin
DAPI
Merge
Interphase
Prophase
Metaphase
Anaphase
Telophase

a

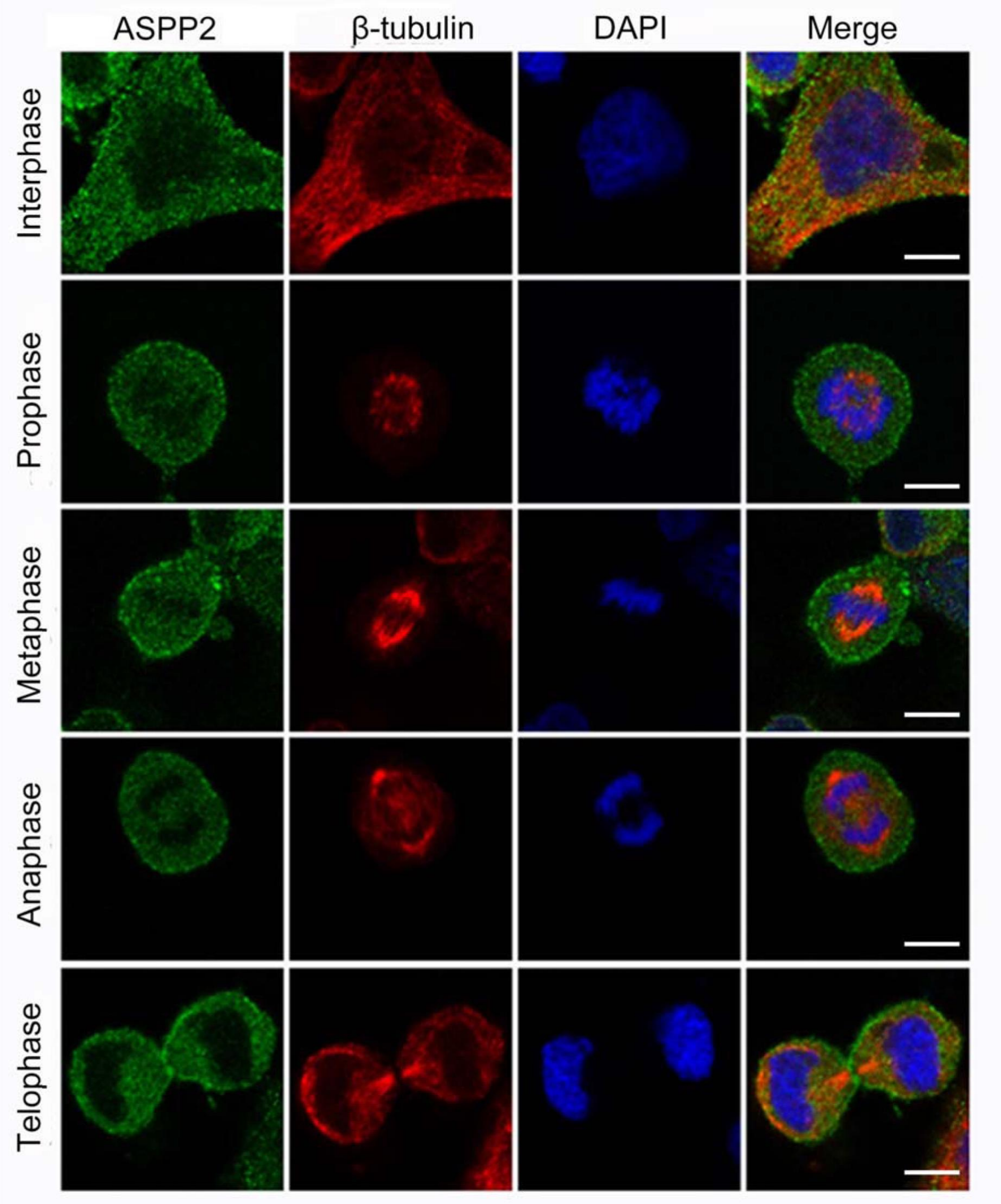
ASPP2
β-tubulin
DAPI
Merge
Interphase
Prophase
Metaphase
Anaphase
Telophase

b

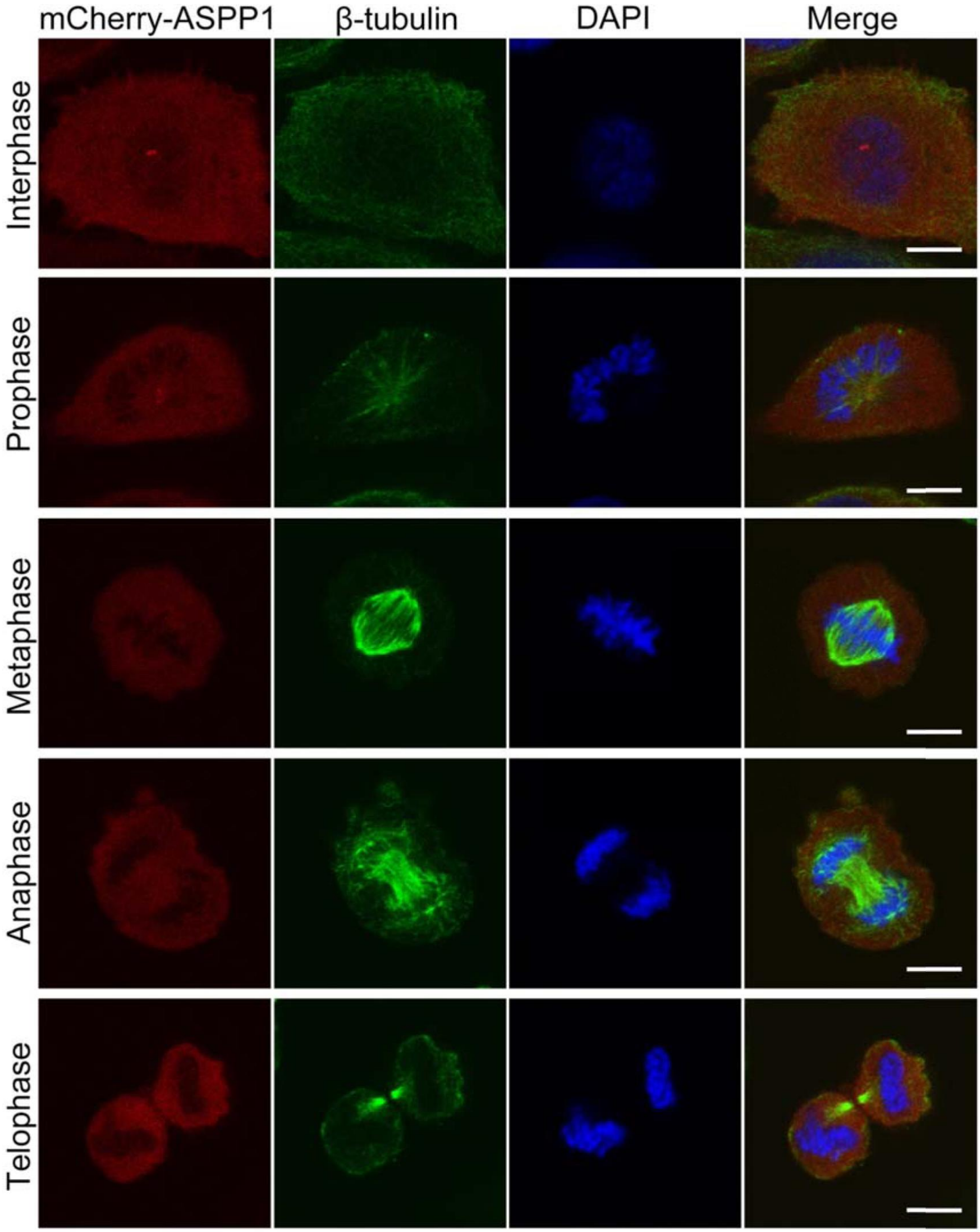
mCherry-ASPP1
β-tubulin
DAPI
Merge
Interphase
Prophase
Metaphase
Anaphase
Telophase

c

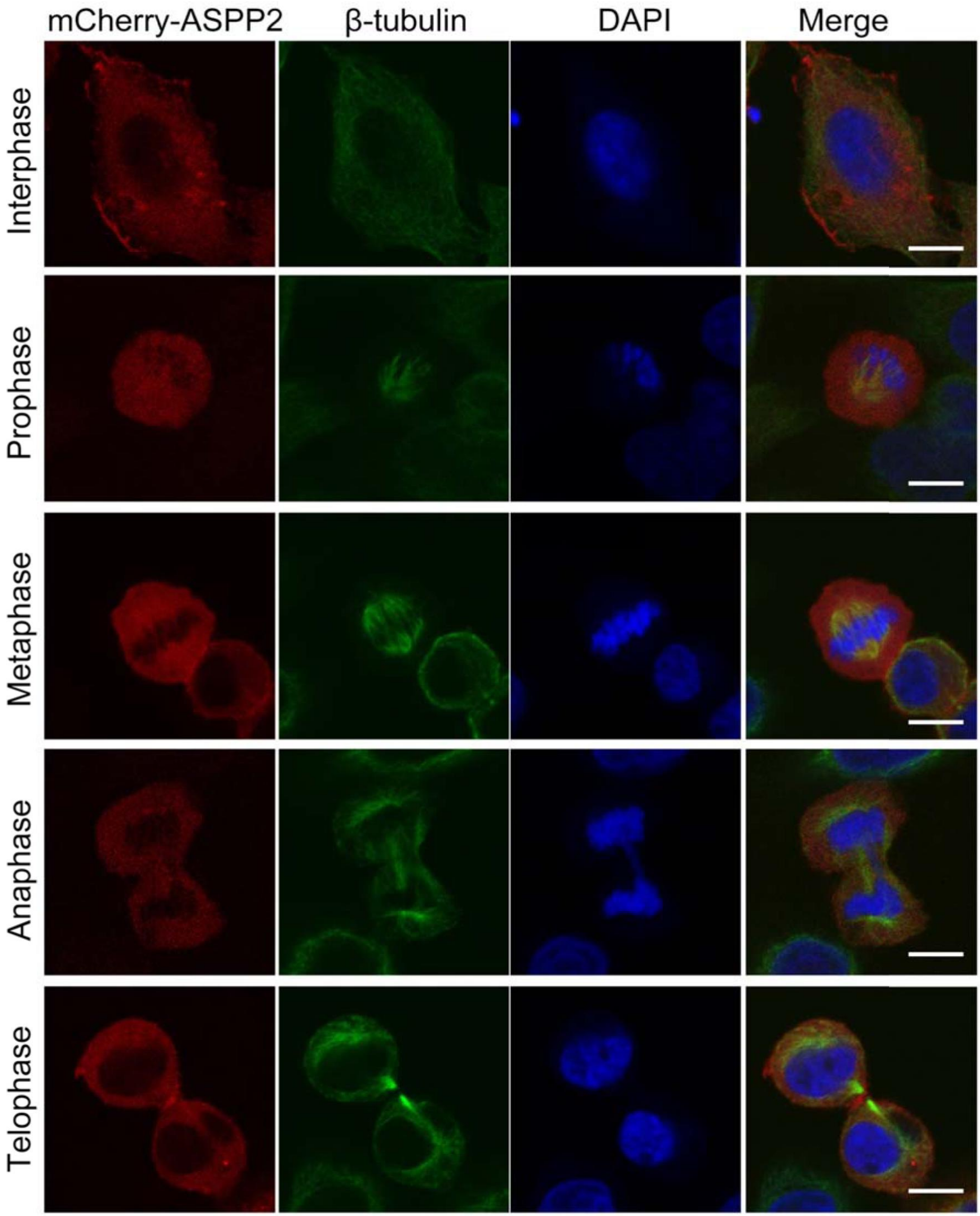

mCherry-ASPP2
β-tubulin
DAPI
Merge
Interphase
Prophase
Metaphase
Anaphase
Telophase

d

Figure S2. Subcellular localization of ASPP1/2 during mitosis. (**a, b**) HeLa cells were stained with anti-ASPP1 or ASPP2 (green), anti-β-tubulin (red) antibodies and DAPI (blue).Scale bar = 10 μm. (**c, d**) Localization of mCherry-tagged ASPP1 or ASPP2 transiently expressed in HeLa cells is shown. HeLa cells were stained with the anti-β-tubulin (green) antibody and DAPI (blue). Scale bar = 10 μm.

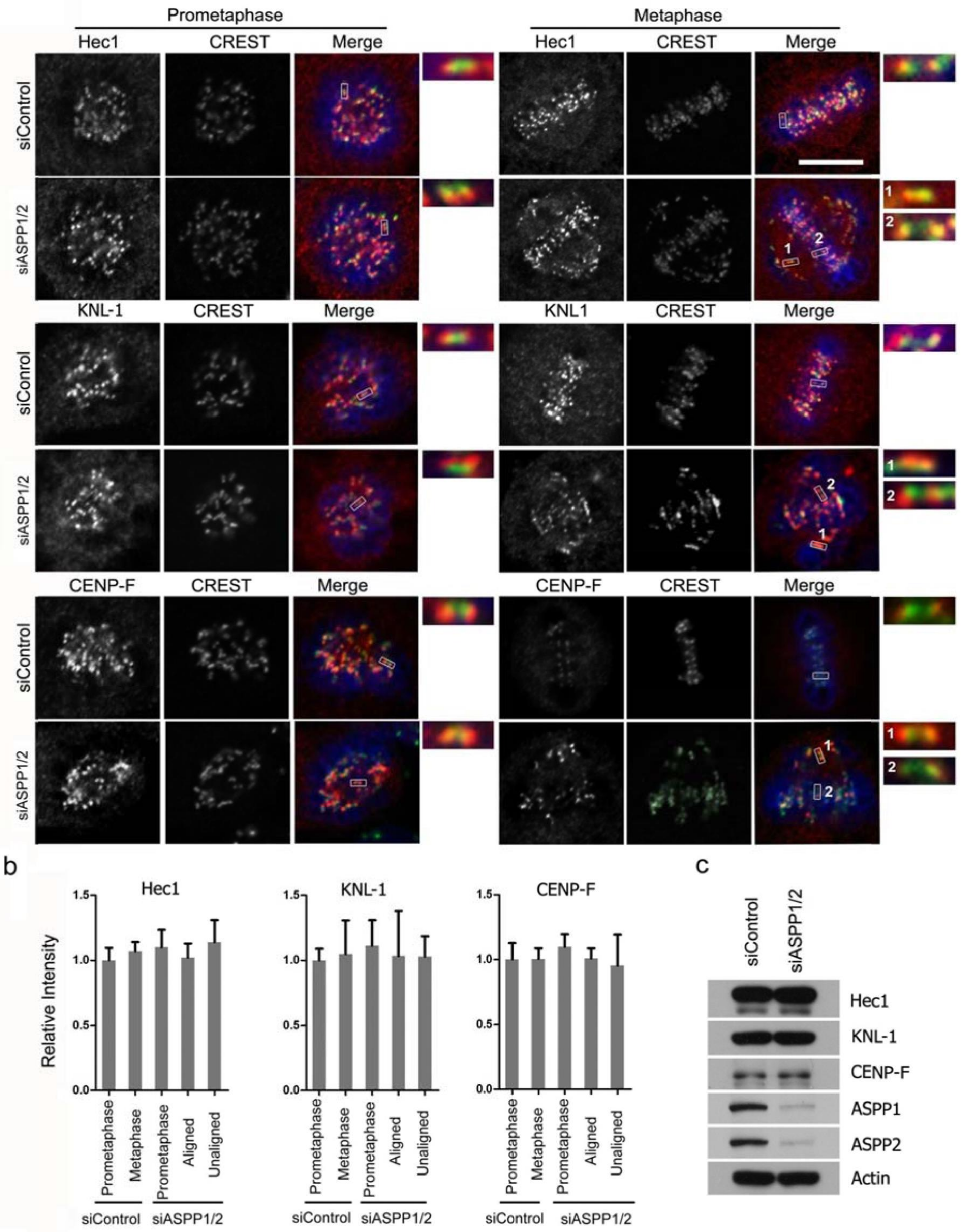

Prometaphase
Metaphase
Hec1
CREST
Merge
siControl
siASPP1/2
KNL-1
KNL1
CENP-F
1
2
b
Hec1
KNL-1
CENP-F
Relative Intensity
1.5
1.0
0.5
0.0
Prometaphase
Metaphase
Aligned
Unaligned
siControl
siASPP1/2
c
Hec1
KNL-1
CENP-F
ASPP1
ASPP2
Actin

Figure S3. ASPP1/2 co-depletion does not alter the kinetochore localization of Hec1, KNL-1 or CENP-F. (**a**) Hec1, KNL-1 or CENP-F localization in ASPP1/2 co-depleted HeLa cells. HeLa cells were transfected with control or ASPP1/2 siRNAs for 48 hr, and with nocodazole for 12 hr and then released into fresh media for 1-2 hr before fixation. Cells were stained for the antibodies against the indicated Hec1, KNL-1 or CENP-F (red), together with kinetochores (CREST, green) and DNA (blue). The figures show confocal images of cells at prometaphase and metaphase. Insets are magnified images of the boxed areas. Scale bar = 10 μm. (**b**) Quantification of the fluorescence intensity of the Hec1, KNL-1 or CENP-F of normalized to the fluorescence intensity of CREST staining are shown. For quantifications, ~30 mitotic cells were measured for each experiment and condition. (**c**) WB analyses of cell lysates prepared from control and ASPP1/2 co-depleted HeLa cells with the indicated antibodies.

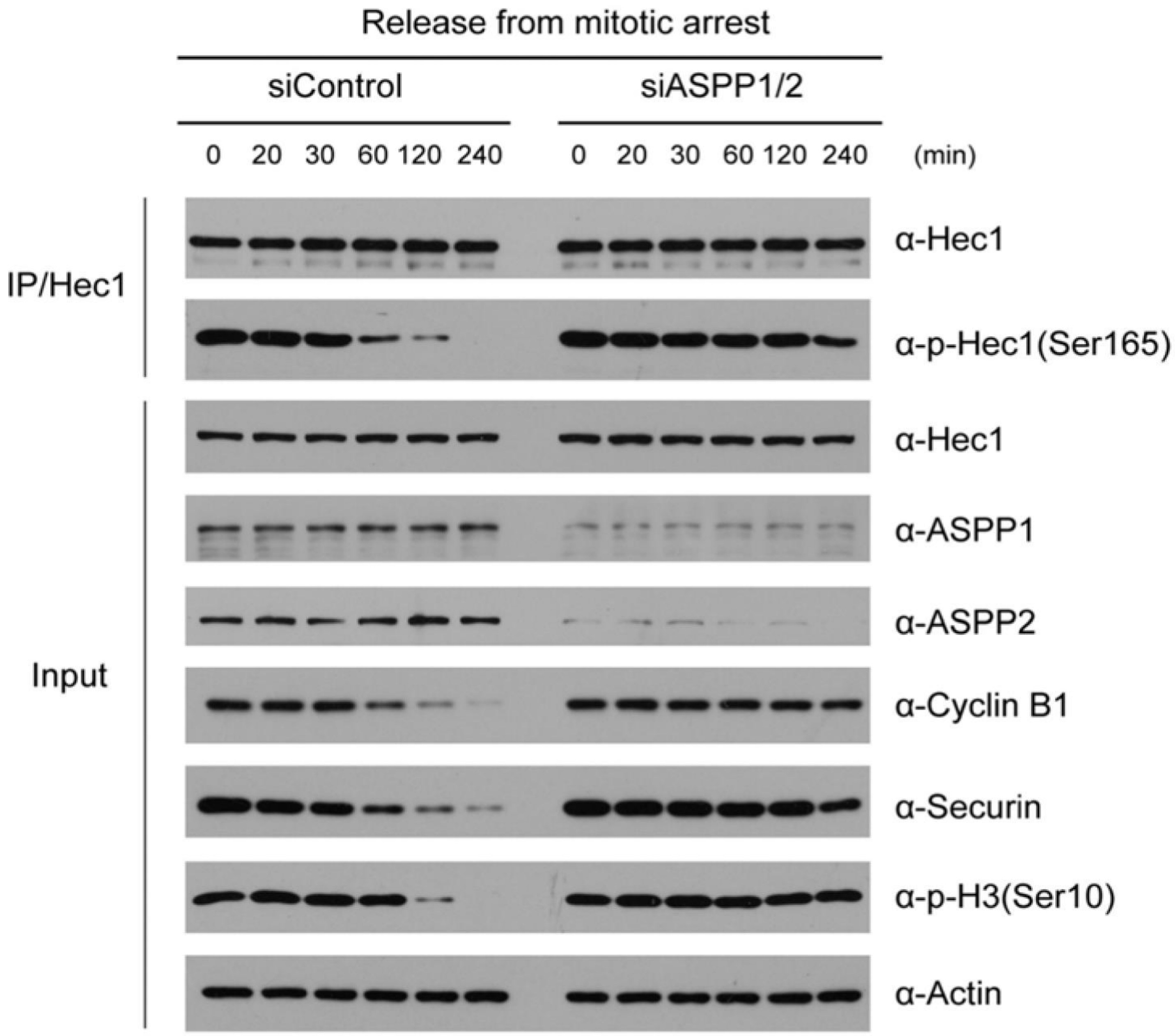

Figure S4. ASPP1/2 co-depletion causes prolonged mitotic exit. HeLa cells were transfected with control or ASPP1/2 siRNAs. After 24 hr, HeLa cells were arrested in prometaphase by a sequential thymidine-nocodazole block and shake off (0 min), or released from a prometaphase arrest by incubation in free media for the indicated times (20-240 min). The cell lysates were prepared for immunoprecipitation with anti-Hec1 antibody and WB analyses with indicated antibodies.
